# Transient Complete Atrioventricular Block During Routine Colonoscopy: A Benign Vagal Reflex Mimicking Conduction System Disease

**DOI:** 10.7759/cureus.99741

**Published:** 2025-12-20

**Authors:** Sebastian Hernandez Mejia, Joud Fahed, Rayna Isber, Nidal Isber

**Affiliations:** 1 Internal Medicine/Cardiology, Richmond University Medical Center, New York, USA; 2 Internal Medicine, Ascension St. Agnes Medical Center, Baltimore, USA; 3 Biology, Barnard College, Columbia University, New York, USA; 4 Electrophysiology, Richmond University Medical Center, Staten Island, USA

**Keywords:** colonoscopy complications, complete heart block (chb), high degree av block, increased vagal tone, mobitz type 2 av block

## Abstract

Complete heart block during gastrointestinal procedures is rare but clinically significant. These episodes are most commonly mediated by excessive vagal stimulation induced by sedation or endoscopic manipulation, and they typically resolve spontaneously without the need for emergent intervention. We report the case of a 68-year-old woman who developed transient complete heart block during routine colonoscopy and upper endoscopy performed under anesthesia. Her rhythm normalized without pharmacologic therapy or pacing. This case underscores the importance of recognizing vagally mediated atrioventricular (AV) block in procedural settings, as misinterpretation of these transient events may lead to unnecessary evaluations or inappropriate permanent pacemaker implantation. Increased awareness of this benign, reflex-mediated phenomenon may help clinicians avoid overdiagnosis of intrinsic conduction disease.

## Introduction

Reflex-mediated cardiac conduction disturbances, particularly those triggered by vagal activation, are well recognized but frequently underappreciated during gastrointestinal procedures. Endoscopic stimulation, visceral distension, and sedative agents can significantly enhance parasympathetic tone, predisposing patients to transient bradyarrhythmic events [1,2]. These may include profound sinus bradycardia, high-grade atrioventricular (AV) block, complete AV block, or brief episodes of asystole [3]. Importantly, these disturbances are functional and reversible, reflecting heightened vagal influence on the sinus and AV nodes rather than intrinsic conduction system disease [4]. Vagally mediated episodes are typically short-lived, with conduction recovering within seconds to a few minutes, and any residual sinus bradycardia usually resolving spontaneously in the immediate post-procedural period.

Bradyarrhythmias most often presents as mild sinus slowing, while high-grade AV block is exceptionally rare and typically brief. Because these episodes are so short, they are rarely recorded clearly and can be misinterpreted as intrinsic conduction disease if the clinical context is overlooked [4]. Recognizing the vagal setting helps avoid unnecessary testing and pacemaker implantation.

We present a case of transient, procedure-related complete heart block that demonstrates the spectrum of vagally induced conduction abnormalities that may occur during routine gastrointestinal endoscopy.

## Case presentation

A 68-year-old woman with hypothyroidism and no known cardiac disease was scheduled for a routine screening colonoscopy in April 2025. Her only medication was levothyroxine 75 μg daily, and she was not taking any AV nodal-blocking agents. Pre-procedure vital signs were within normal limits, and a 12-lead electrocardiogram showed normal sinus rhythm without conduction abnormalities; her body mass index was 17 kg/m². Bowel preparation was adequate, a pediatric colonoscope was used, and CO₂ insufflation was maintained throughout the procedure with good mucosal visualization. Despite these favorable conditions, the colonoscopy was technically difficult because of a tortuous sigmoid colon, requiring repeated scope withdrawal and reduction maneuvers to advance to the cecum.

About five to seven minutes into the manipulation of the sigmoid colon, the monitor first showed sinus bradycardia at around 50 beats/min, followed by an abrupt drop in heart rate to 22 beats/min. A lead II rhythm strip captured a long ventricular pause with loss of AV conduction, consistent with a transient high-grade AV block, after which the rhythm reverted to marked sinus bradycardia. Blood pressure remained stable at 110/60 mmHg. The patient remained deeply sedated and did not manifest chest pain, dyspnea, or presyncope. Normal sinus rhythm returned spontaneously within approximately 60 seconds without pharmacologic therapy or pacing. 

**Figure 1:**
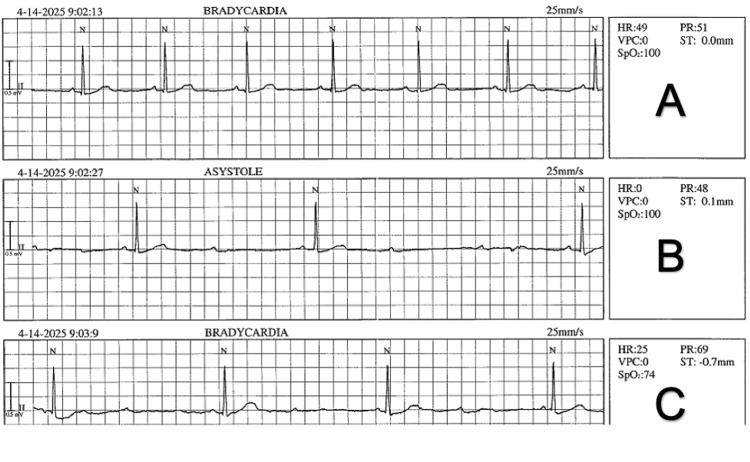
Rhythm strip - spectrum of vagal-mediated conduction changes during colonoscopy A. Initial stable sinus bradycardia at ~50 bpm. B. Sudden long ventricular pause consistent with transient complete AV block. C. Intermittent non-conducted P waves (a brief high-grade/Mobitz II–type pattern) before settling into marked sinus bradycardia. AV: Atrioventricular

While the full duration of complete AV block was not preserved in the saved telemetry strip, the available printed tracings (Figure 1) clearly illustrate the clinical sequence: stable sinus bradycardia, an abrupt transition to high-grade AV conduction delay with a significant ventricular pause, and the subsequent profound sinus bradycardia. The complete third-degree AV block observed in real time occurred immediately before the recorded segment and was not fully captured on paper. Despite this transient conduction disturbance, colonoscopy was completed to the cecum without further arrhythmia or hemodynamic instability.

Following the event, she remained hemodynamically stable and was immediately referred for electrophysiology (EP) assessment. At the time of evaluation by the EP service, her vital signs were normal (blood pressure 120/70 mmHg, heart rate 76 beats/min), and physical examination was unremarkable. She reported no history of syncope or presyncope, although she recalled occasional mild dizziness over the past several years. Review of prior records revealed a screening colonoscopy performed under propofol anesthesia that had been technically straightforward and uneventful, without documented rhythm disturbances. She denied tobacco or alcohol use, and there was no family history of arrhythmias, conduction disease, or pacemaker implantation.

A post-procedure 12-lead ECG demonstrated normal sinus rhythm with normal PR, QRS, and QT intervals, supporting the impression of a reversible, vagally mediated conduction disturbance rather than intrinsic conduction system pathology. At outpatient EP follow-up after the procedure, she remained asymptomatic without syncope, presyncope, or exertional symptoms. No recurrent bradyarrhythmias or AV block were documented, and pacemaker implantation was not considered necessary.

## Discussion

This case illustrates a classic example of transient, vagally mediated complete AV block occurring during gastrointestinal endoscopy. These reflex-mediated episodes result from abrupt increases in parasympathetic activity, which exert strong inhibitory effects on the sinoatrial (SA) and AV nodes-structures richly innervated by vagal fibers from the dorsal motor nucleus and nucleus ambiguus [1].

At the cellular level, acetylcholine released at M2 muscarinic receptors activates G-protein-coupled inward-rectifier potassium channels (GIRK/IKACh), producing nodal hyperpolarization, sinus slowing, and prolongation of AV nodal conduction time [2]. When vagal activation is intense, this can transiently interrupt all AV conduction, producing a short, fully reversible episode of complete heart block, typically without involvement of the His-Purkinje system [3].

Gastrointestinal procedures are well-recognized triggers of this reflex. Manipulation of the pharynx, esophagus, rectum, or rapid colonic insufflation can activate vagal afferent pathways, producing abrupt cardioinhibitory responses. Experimental work has shown that visceral stretch and mechanosensation can activate specific vagal sensory neuron populations capable of inducing bradycardia, hypotension, or AV block [4]. Reviews of vagal neurophysiology reinforce how rapid acetylcholine release can transiently suppress AV nodal conduction even in structurally normal hearts [5]. In endoscopic practice, common triggers include air insufflation, mesenteric stretch, or scope advancement. Esophageal intubation has been shown to provoke significant vagal slowing, while peritoneal stretch during pneumoperitoneum can reproduce similar responses [6,7].

Sedative choice can further influence this vagal susceptibility. Among commonly used agents, propofol, the sedative used in this case, appears to carry the highest risk of transient bradyarrhythmias and high-grade AV block because it enhances vagal tone and slows sinus and AV nodal conduction. Dexmedetomidine confers an intermediate risk, promoting bradycardia and AV nodal suppression via central sympatholysis, with reported cases of transient high-grade AV block [8]. Opioids such as fentanyl may augment vagal tone and contribute to sinus bradycardia, whereas midazolam alone is generally considered lower risk for clinically significant conduction abnormalities; however, synergistic events can occur, particularly during painful or nocturnal procedures when baseline vagal tone is higher [9].

Several studies have specifically examined rhythm changes during colonoscopy. George et al. found that bradyarrhythmias during colonoscopy were primarily vagal in origin and almost always self-limited, with no patients requiring invasive intervention [10]. Arrowsmith et al., in a prospective study, reported that rhythm disturbances, including sinus slowing and occasional AV nodal delay, were not uncommon during endoscopy but rarely progressed to clinically significant arrhythmias or required treatment [11]. Vazharov similarly reported a spectrum of rhythm abnormalities during colonoscopy, noting that most episodes were vagally mediated, transient, and resolved without long-term sequelae [12]. Sedation-focused reviews in gastrointestinal endoscopy further emphasize that vagal bradyarrhythmias in this setting are typically brief and self-limited, even when moderate or deep sedation is used [13].

In these colonoscopy series, most documented bradyarrhythmias were attributed to the same triggers already discussed, painful colonic distension, looping, and traction rather than to scope size or insufflation modality, and none systematically compared adult versus pediatric scopes or air versus CO₂ [10,12,13]. Together, these data support the view that mechanical factors, with vagotonic sedation, are the dominant drivers of reflex bradyarrhythmias during endoscopy, and that while mild bradycardia is relatively common, true high-grade or complete AV block is exceptionally rare and typically benign. Supportive measures such as pausing the procedure, reducing insufflation, or administering IV fluids are usually sufficient [14]. 

In this patient, the sudden onset of complete AV block immediately following endoscopic manipulation, along with spontaneous recovery and a completely normal post-event ECG, strongly supports a functional, vagally mediated disturbance. She had no prior symptoms or conduction abnormalities, and her PR, QRS, and QT intervals were normal after recovery. These features-along with a clear procedural trigger and rapid resolution-are characteristic of benign vagal block rather than intrinsic conduction system disease.

Distinguishing reflex-mediated AV block from intrinsic AV nodal or His-Purkinje disease is essential because management differs significantly. Reflex-mediated episodes are abrupt in onset, brief, and associated with sinus slowing; they resolve with removal of the stimulus. In contrast, intrinsic conduction disease is often associated with baseline ECG abnormalities or recurrent symptoms and may not respond to atropine. The 2018 European Society of Cardiology (ESC) Syncope Guidelines emphasize careful assessment of clinical context to avoid unnecessary pacemaker implantation in cases of clearly vagal origin [15].

Our patient’s clinical course aligns closely with these observations. She experienced abrupt complete AV block during a known vagal stimulus, followed by spontaneous normalization of rhythm and ECG parameters. She had no prior history of conduction disease, no recurrent symptoms, and no persistent abnormalities after the event. Recognition of this pattern prevented unnecessary intervention, including pacemaker implantation. In patients without recurrent episodes or evidence of intrinsic conduction disease, no additional evaluation is typically required.

## Conclusions

Vagally mediated bradyarrhythmias may occur during gastrointestinal endoscopy, usually in response to triggers such as colonic distension, looping, or traction. This case highlights how a pronounced vagal reflex during routine colonoscopy can, on rare occasions, temporarily produce complete AV block in a patient with an otherwise normal heart. The brief, self-terminating nature of the episode, together with a normal post-procedure ECG and absence of prior symptoms, supports a benign, vagally mediated mechanism rather than intrinsic conduction system disease. Recognizing this pattern is essential to avoid unnecessary invasive evaluation and pacemaker implantation. In patients with a clear procedural trigger and complete recovery, conservative management with observation and reassurance is usually sufficient.
